# Tolerating the Unwelcome Guest; How the Host Withstands Persistent *Mycobacterium tuberculosis*

**DOI:** 10.3389/fimmu.2018.02094

**Published:** 2018-09-12

**Authors:** Andrew J. Olive, Christopher M. Sassetti

**Affiliations:** ^1^Department of Microbiology and Molecular Genetics, Michigan State University, East Lansing, MI, United States; ^2^Department of Microbiology and Physiological Systems, University of Massachusetts Medical School, Worcester, MA, United States

**Keywords:** *Mycobaterium tuberculosis*, tolerance, inflammasome, immunometabolism, persistent infections

## Abstract

Our understanding of the host response to infections has historically focused on “resistance” mechanisms that directly control pathogen replication. However, both pathogen effectors and antimicrobial immune pathways have the capacity to damage host tissue, and the ability to tolerate these insults can also be critical for host survival. These “tolerance” mechanisms may be equally as important as resistance to prevent disease in the context of a persistent infection, such as tuberculosis, when resistance mechanisms are ineffective and the pathogen persists in the tissue for long periods. Host tolerance encompasses a wide range of strategies, many of which involve regulation of the inflammatory response. Here we will examine general strategies used by macrophages and T cells to promote tolerance in the context of tuberculosis, and focus on pathways, such as regulation of inflammasome activation, that are emerging as common mediators of tolerance.

## Introduction

The ultimate goal of the host response to bacterial pathogens is to survive the infection. Much of the research to understand protective immunity has historically had a singular focus on antimicrobial resistance mechanisms that directly control bacterial replication. In general, these “resistance” mechanisms act by poisoning the pathogen, disrupting the pathogen's niche, or sequestering nutrients in an attempt to restrict growth and spread (1, 2). Classic resistance pathways include the antimicrobial peptide production from epithelial surfaces and the microbicidal functions of phagocytes which are augmented by antigen-specific lymphocyte responses. More recently it has become clear that in addition to these resistance strategies, the host also relies on distinct mechanisms that allow it to withstand infections independently of controlling bacterial growth (3, 4). These “tolerance” mechanisms represent host pathways that modulate diverse aspects of physiology. Both the local control of inflammatory tissue damage and repair, as well as systemic responses such as anorexia, and fever have been shown to promote host survival in a number of infection contexts (5, 6). For many self-resolving infections, resistance mechanisms may be sufficient to restrict bacterial replication and minimize pathology (2). However, some pathogens, like *Mycobacterium tuberculosis* (*Mtb*), are able to resist many of the resistance mechanisms of the host and persist for long periods (7). In these situations, tolerance pathways are critical for preventing the progressive pathology elicited by the persistent presence of the pathogen. Tolerance responses ensure that the locally infected tissues continue to function and that the overall health of the host is maintained (3, 8).

While potential therapies that promote host resistance have received a great deal of interest, promoting tolerance pathways that decrease morbidity and/or mortality in the face of an ongoing chronic infection could represent an equally appealing avenue for intervention (9, 10). In this review, we will discuss the host response to *Mtb* infections from the viewpoint of host tolerance. While tolerance encompasses a potentially large array of host functions, we will consider known and emerging mechanisms that limit lung damage and discuss how distinct cell populations like macrophages and T cells contribute to tolerance by controlling cytokine production and metabolic functions. Ultimately, understanding host tolerance mechanisms will define new pathways of protective immunity to tuberculosis (TB), and could identify new therapeutic strategies.

### Tuberculosis pathogenesis

*Mtb* infections are transmitted by aerosol (7, 11). Following inhalation of contaminated droplets, *Mtb* is engulfed by alveolar macrophages, where the pathogen replicates and evades the innate antimicrobial mechanisms of this cell (7, 11, 12). After the activation of host adaptive immune responses, bacterial growth is slowed or halted. While evidence from non-human primates (NHP) and human autopsy studies indicate that some infectious foci can be sterilized, the pathogen is able to persist in the face of this adaptive response for long periods. In some individuals, this infection produces the chronic inflammatory disease called, tuberculosis (TB). While any organ in the body can be affected, pulmonary disease promotes transmission of the pathogen, beginning a new infectious cycle.

For most individuals, chronic infection with *Mtb* does not produce symptomatic disease (7, 13). However, a subset of individuals (5–10%) will progress to develop TB after a period of asymptomatic infection that generally lasts for less than 2 years, but can extend for decades in rare cases (14, 15). What drives the heterogeneity of disease progression is not entirely known and is likely a combination of host and bacterial genetic diversity, as well as environmental factors (3, 8, 16). Several distinct aspects of TB pathogenesis could be affected by host tolerance pathways. Most obviously, the risk of developing disease is likely to depend on host tolerance. Most infected individuals never develop symptoms, and the ability to harbor this immunogenic pathogen for long-periods without suffering from progressive pathology likely depends on the ability to control inflammation(10, 17, 18). In fact, the phenomenon of “latent TB infection” (LTBI) could be considered one of the clearer examples of pathogen tolerance in humans. Patients that are cured of TB by antibiotic therapy suffer from reduced respiratory function, indicating that even after bacteria are eradicated, local tissue damage persists (19–21). In fact, multiple rounds of infection and antibiotic therapy are associated with increased erosion of lung function (21). This effect is not simply additive, as rabbits exposed to 5 sequential low dose infections developed significantly more severe cavitary disease than animals exposed to a single large dose of *Mtb* (22). Thus, tolerance mechanisms that control local tissue damage could determine long-term outcome and are influenced by environmental factors such as the frequency of infection. Manifestations of *Mtb* other than pulmonary disease may be even more dependent on host tolerance mechanisms that control inflammation (23). For example, meningeal *Mtb* infection is associated with very high mortality, which is related to the expression of inflammatory cytokines (24, 25). Similarly, TB immune reconstitution inflammatory syndrome (TB-IRIS) is a condition that occurs in HIV/*Mtb* co-infected individuals soon after starting antiretroviral therapy (26). This syndrome still results in almost 40% mortality, and is associated with failed regulation of inflammatory cascades (27–30).

The mechanisms that control TB tolerance are complex because interactions between multiple cell types influence disease progression. Following infection and activation of the host immunity, infected cells are walled off in large structures termed a granuloma (7, 16). Granulomas are thought to be required for the host to tolerate *Mtb* infections, yet their development and progression throughout infection may also drive *Mtb* survival and transmission. Bacterial barcoding and PET-CT studies in non-human primates have shown individual granuloma that are formed from single founder bacteria can have very distinct fates, some contain the pathogen and while others progressively develop into the large cavities that typify pulmonary TB disease (16, 31, 32). As a result, individual lesions are variable in their disease trajectories and transmission potential suggesting complicated dynamics determine the outcome of each lesion (31, 32). Beyond granuloma development, influx of leukocytes such as neutrophils and the expression of proteases such as matrix metalloproteinases (MMPs) can reduce host tolerance by irreversibly damaging tissue (33, 34). As the role of MMPs and neutrophils in modulating immunopathology to *Mtb* have been reviewed elsewhere, we will focus on how macrophages and T cells modulate host tolerance to determine the outcome of *Mtb* infections (35, 36).

### Macrophages and tolerance

Macrophages are an important intracellular niche for *Mtb* to replicate yet they can also restrict *Mtb* growth in an activation dependent manner (12). The balance between *Mtb* replication and control is determined by a diverse array of resistance pathways, including those activated by interferon-γ (IFNγ), granulocyte-macropahge colony stimulating factor (GM-CSF) and interleukin-1β (IL-1β) (37–39). Due to their direct interactions with *Mtb*, macrophages are also central regulators of host tolerance. Several lines of evidence suggest that tolerance mechanisms modulated by macrophages may play a significant role in determining disease progression and controlling the outcome to *Mtb* disease.

#### Nitric oxide

**Figure 1:**
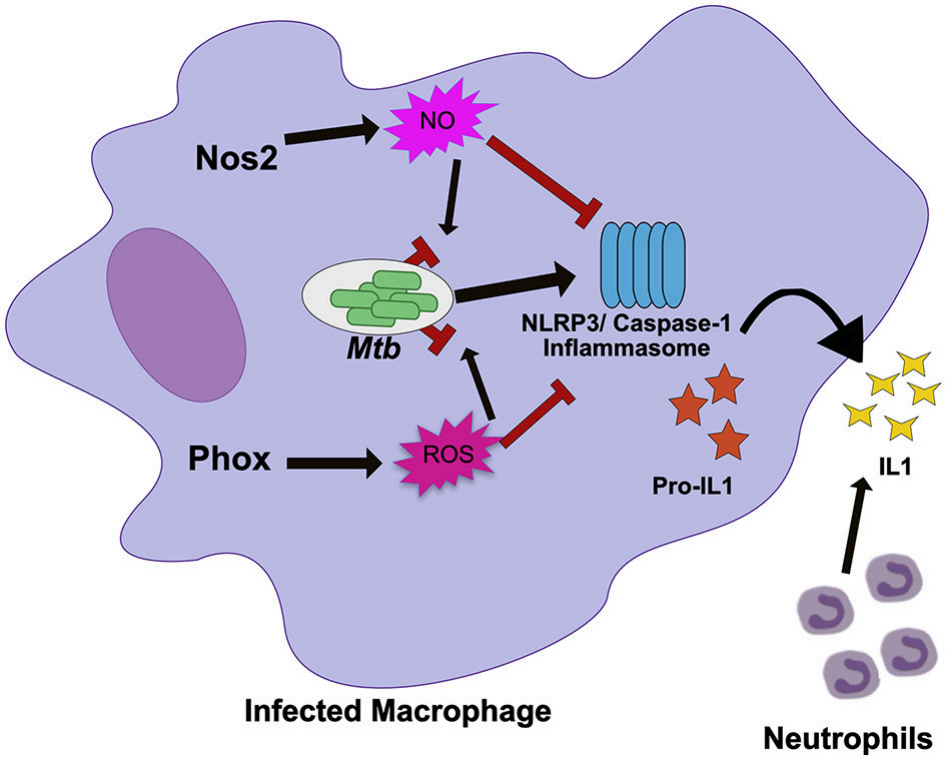
Nos2 and Phox control tolerance during Mtb infection by negatively regulating inflammasome activation. During *Mtb* infection in macrophages, Nos2 and Phox produce NO and ROS respectively. While these molecules are antimicrobial against many pathogens, *Mtb* is mostly resistant. Persistent *Mtb* then activates the NLRP3 inflammasome to produce active IL1β. Prolonged inflammasome activation leads to increased IL1β secretion and neutrophil recruitment that damages the lungs. In order to tolerate persistent infections with *Mtb*, the NO and ROS produced by macrophages also suppresses inflammasome activation to limit the damage caused by recurring neutrophil recruitment. NO directly nitrosylates NLRP3 while the mechanisms of ROS inhibition remain unknown.

One compelling case for the role of tolerance in macrophages during chronic *Mtb* infections is that of inducible nitric oxide synthase (Nos2). For years, it was generally presumed that the protective function of Nos2 could be attributed to the direct antimicrobial activity of nitric oxide (NO) (40). In support of this hypothesis was data that showed that Nos2 deficient mice are extremely susceptible to *Mtb* infection (40, 41). These animals die within 2 months of infection with 10–100 fold more bacteria in lungs than wild type animals as well as a massive infiltration of tissue-damaging neutrophils. Recent evidence however suggests that the situation is more complex. Mtb expresses a number of defense mechanisms that protect the pathogen from the antimicrobial effects of NO, and recent evidence suggests that the role of Nos2 in regulating inflammatory pathways and host tolerance play a dominant role in protection (Figure 1) (42–45).

Disentangling tolerance pathways *in vivo* is a significant challenge due to the interlinked nature of bacterial load and tissue damage; higher bacterial burdens can lead to more inflammation and tissue damage, while higher tissue damage and inflammation may create an environment that drives more bacterial replication (8). The role of each host effector in controlling resistance or tolerance pathways may also be timing and context dependent (46). It is also likely that many pathways control both resistance and tolerance during persistent infection (47). Because of this, distinct *in vivo* models that control either inflammation or bacterial replication are required to break down the mechanisms of a particular “protective” gene like *Nos2*. When these models were applied to Nos2, it became clear in Nos2 deficient animals succumb to *Mtb* infection through hyperinflammatory disease, even when bacterial load is controlled using a conditionally-replicating strain of the pathogen (43, 44). Subsequent mechanistic studies determined that NO nitrosylates the inflammasome component NLRP3, which inhibits the production of bioactive IL-1β and prevents persistent neutrophil recruitment (44). Similarly, Nos2 has also been shown to dampen the inflammatory response by limiting the activation of NF-κβ (48).

Nos2 serves as an important example of the need to understand the mechanisms by which individual immune effectors protect against TB disease progression. While a modest role for Nos2 in modulating *Mtb* replication in macrophages remains possible, the recent evidence strongly suggests the predominant role of NO production in mice during *Mtb* is to control tolerance by dampening inflammatory pathways.

#### NADPH phagocyte oxidase

Many immune mediators have similarly pleiotropic effects as Nos2, raising the possibility that other well-characterized pathways may also play unanticipated roles in regulating tolerance. The NADPH Phagocyte Oxidase Complex (Phox) provides another example. This system is required to produce a burst of reactive oxygen species (ROS) that intoxicate the intracellular bacteria. The importance of Phox in protecting the host during *Mtb* infections is generally considered minimal because Phox deficient animals show no long-term defects in controlling *Mtb* growth and *Mtb* is equipped with many strategies to resist ROS-mediated killing (41, 49–51). However, human studies suggest that mutations in Phox, which leads to the condition known as chronic granulomatous disease, are associated with higher susceptibility to mycobacterial infections including TB (52, 53). In other disease contexts Phox deficiencies have been found cause inflammatory disease, particularly those related to IL-1β activation (54). Recent work shows that Phox is also critical for tolerance to *Mtb* infection (Figure 1) (55). Phox-deficient mice have no deficiency in bacterial control, yet Phox-deficient animals accumulate high numbers of neutrophils in an IL-1β dependent fashion, leading to exacerbated disease (55). Similar to the role of Nos2, the ROS produced by Phox control tolerance by inhibiting the activation of the NLRP3 inflammasome which reduces IL-1β production and limits neutrophil influx to the infected lung. The fact that the important tolerance-regulating functions for both Nos2 and Phox were overlooked for some time, suggests that tolerance-regulating roles may still be found for additional host response pathways.

The similar ability of Nos2 and Phox to control inflammasome activation suggests that preventing persistent IL-1β production is a common strategy used by the host to tolerate persistent infections. In support of this, human studies have found that altered IL-1β expression modulates TB disease severity (56). IL-1β alleles that enhance IL-1β expression are associated with increased risk of developing TB disease, more severe pulmonary disease, and poor treatment outcome (56). In addition, inflammasome activation is associated with the development of TB-IRIS and TB meningitis (57, 58). Two recent studies suggest that expression and activation of inflammasome components including NLRP3 and the high expression of IL-1β in plasma and the nervous system are signatures of failed tolerance during antiretroviral treatment and a major risk factor to developing fatal disease (57, 58). The repeated association with inflammasome activity, IL-1β production and more severe TB-related pathology suggests that this pathway could serve as a therapeutic target, particularly for the severe inflammatory syndromes with poor outcomes.

#### Lysosomal function and autophagy

Proper maintenance of cellular organelles is important to tolerate *Mtb* infections (59–62). Loss of critical homeostatic pathways can lead to cellular dysfunction and misregulation of inflammatory cytokines during *Mtb* disease. Mycobacterium infections of zebrafish with mutations in cathepsins leads to loss of granuloma integrity and reduced survival due to improper breakdown in lysosomal contents (59). In humans, this mutation is phenocopied in individuals who smoke tobacco. *Mtb* infected macrophages from smokers accumulate particulates in their lysosomes, inhibiting their function and likely altering tolerance. It is well known that previous smoking history can increase the risk of developing TB disease by over two-fold and it is possible that alterations to lysosomal function are a key aspect to these patients TB susceptibility (63).

Autophagy is another key pathway that maintains the integrity of organelles and regulates a variety of important immune-related processes (64). Recently, the role of autophagy in antimicrobial resistance during *Mtb* has been questioned but the importance of Atg5 in tolerance is undeniable (61, 65, 66). Mice with mutations in most autophagy genes control *Mtb* disease normally (61). However, Atg5-/- mice show a unique susceptibility to TB disease. Infection of Atg5 mice leads to a hyperinflammatory disease state with massive neutrophil migration to the pulmonary tissue and rapid mortality (61). Depletion of neutrophils alone in infected Atg5 deficient mice can reverse the susceptibility and allow long term survival arguing against an inherent defect in antimicrobial control. Exactly how Atg5 controls the inflammatory response, or why loss of Atg5 and not other autophagy components drives neutrophil-mediated disease remains to be understood. But it is clear that altering macrophage homeostasis directly modulates tolerance to *Mtb*.

#### Macrophage metabolism

Recent evidence suggests that macrophage metabolic pathways and byproducts can modulate the inflammatory pathways both locally and systemically (1, 2). Similarly, *Mtb* infections are influenced by systemic metabolic dysfunction such as diabetes, which can alter the activation state of macrophages at the site of infection (67). Evidence for how essential local and systemic metabolic networks influence host tolerance to *Mtb* is beginning to emerge.

Central regulators of host cell metabolism are intimately linked with control of inflammatory circuits (68). These pathways, including mammalian target of rapamycin (mTOR), silent mating type information regulation 2 homologs (Sirtuins), and adenosine monophosphate-activated protein kinase (AMPK), are known to regulate cellular functions such as autophagy, NF-kb signaling, and central metabolism. Importantly, many of these networks are disrupted during *Mtb* infection suggesting that they could play a role in regulating the inflammatory milieu that is activated during *Mtb* infection and likely influence host tolerance (69). Because FDA approved modulators of these metabolic networks are available, they represent appealing targets for host directed therapies that may enhance tolerance during *Mtb* infections and improve clinical outcomes (70).

**Figure 2:**
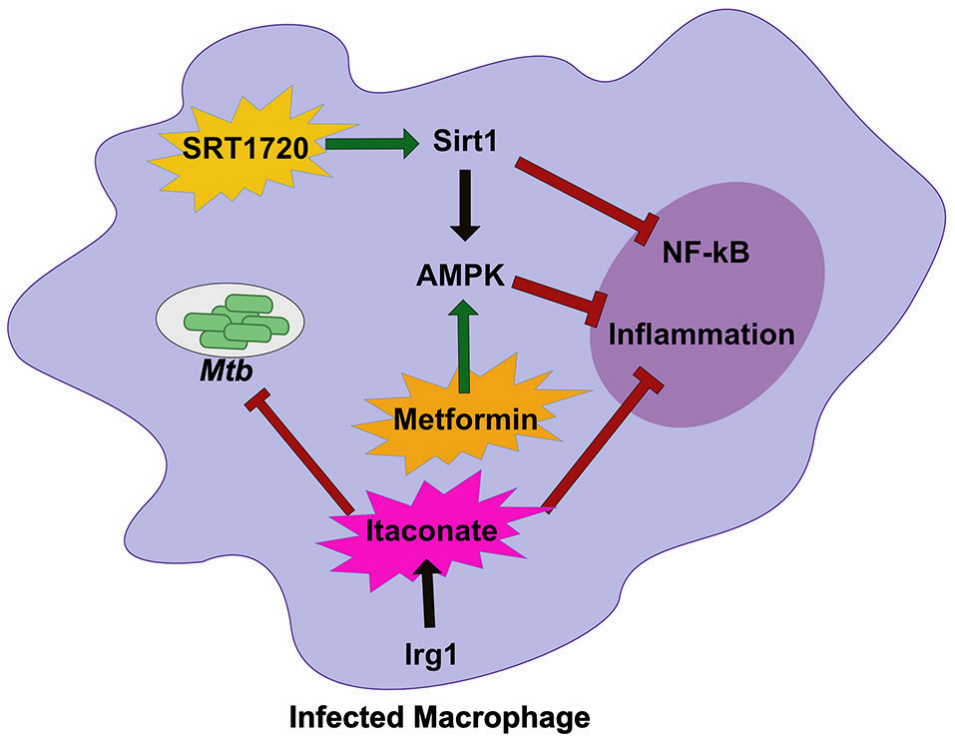
Host metabolic networks modulate tolerance to Mtb infections. Distinct metabolic networks control the inflammatory response during *Mtb* infection. Small molecule activation of Sirt1 with SRT1720 inhibits NF-κβ signaling and activates AMPK and promote tolerance to *Mtb*. This is similar to treatment with the diabetes drug Metformin that activates AMPK to inhibit inflammation and allow the host to better tolerate persistent *Mtb* infections. An alternative metabolic network activated by Irg1, produces the metabolite Itaconate. Itaconate can directly restrict *Mtb* replication, but *in vivo* robustly controls tolerance by modulating the inflammatory response to persistent infection. Together these metabolic networks directly and indirectly control tolerance to *Mtb* infection.

Sirtuin 1 (SIRT1), a known regulator of host stress responses, is downregulated during *Mtb* infection (71). In order to understand how the loss of SIRT1 function impacts *Mtb* disease, Singhal and colleagues treated infected macrophages and animals with a known small molecule SIRT1 activator (Figure 2) (71). While activation of SIRT1 resulted in a modest reduction in bacterial growth *in vitro* and *in vivo*, it led to dramatic changes in the inflammatory profile of infected macrophages and immunopathology in mice, indicating that activation of SIRT1 promotes host tolerance during *Mtb* infection. Interestingly, SIRT1 activation during *Mtb* results in similar outcomes to treatment with the AMPK activator metformin, a common treatment for diabetes (72). During *Mtb* infection, metformin treatment leads to subtle decreases in bacterial burden but larger decreases in inflammatory cytokines and tissue damage (Figure 2). A retrospective study of diabetic TB patients indicates that metformin may improve outcomes. SIRT1 can also influence AMPK signaling, suggesting that the SIRT1/AMPK signaling axis may be a critical regulator of tolerance during *Mtb* infection. It is also intriguing that diabetes treatments such as metformin, are so effective against treating *Mtb* disease. Diabetes increases *Mtb* risk in humans (73, 74). In a mouse model of hyperglycemia, there was a profound effect on neutrophil accumulation during *Mtb* infection which worsened disease outcome (75). Thus, while it likely the complex effect of diabetes on immunity could include resistance defects, in the mouse model tolerance defects appear to dominate.

Another important metabolic pathway that modulates tolerance to *Mtb* is mediated by the mitochondrial enzyme immune responsive gene 1 (Irg1) (76). Irg1 produces the metabolite itaconate that recently was shown to dampen cytokine production and reduce damaging ROS during *Mtb* infection (Figure 2) (77). Loss of Irg1 *in vivo* leads to rapid mortality that is driven by hyper-inflammation and neutrophil-mediated disease. Itaconate alone is sufficient to reverse the increase in pro-inflammatory gene expression in infected Irg1 deficient macrophages suggesting this metabolite is a robust regulator of tolerance to *Mtb*. While itaconate can directly inhibit bacterial growth, *in vivo* studies indicate that its immunomodulatory function may play a dominant role (76, 77). Future studies will need to carefully dissect the role of Irg1 in both controlling resistance and tolerance to fully understand its pleiotropic functions during *Mtb* infection.

### T cells and tolerance

T cells are critical for resistance to *Mtb* (7, 78). In addition, it is clear that Th1 cells that produce IFNγ promote tolerance by activating the production of NO and by directly inhibiting the recruitment of neutrophils (44, 79). This profound effect on *Mtb* protection suggested that more robust activation of Th1 cells would lead to improved disease outcomes. In reality, the situation is much more complicated and recent evidence suggests that activating enhanced Th1 responses to *Mtb* leads to increased susceptibility through failed tolerance.

*IFN*γ. The cytokine IFNγ is produced by activated T cells during *Mtb* infection and is essential for protection of the host. During chronic infections, the levels of IFNγ produced by individual T cells can wain due to persistent antigen and T cell exhaustion (80). Targeting inhibitory receptors on T cells might drive enhanced cytokine responses and lead to more robust *Mtb* control. As a proof of principle of this concept mice lacking the T cell inhibitory receptor PD1 were infected with *Mtb* (81). Surprisingly, rather controlling *Mtb* infection better, PD1 deficient animals had decreased tolerance that was characterized by increased susceptibility and immunopathology. This counterintuitive result suggested that more robust T cell responses might be detrimental to long term *Mtb* protection. What is driving the decrease in tolerance in these animals? One recent study began to examine the mechanisms modulating the tolerance defect in PD1 deficient mice and showed that increased IFNγ production is responsible (82). When PD1 deficient T cells no longer make IFNγ, the defect in tolerance is reversed. In addition, CD4^+^ T cells that produce more IFNγ on a per cell basis do not control *Mtb* growth more effectively in the lungs, but rather cause tissue damage and more rapid mortality. Similarly, T cells with mutations in the Calcium channel ORAI1 activating protein Stim1 are unable to undergo apoptosis following infection leading to a significant increase in IFNγ in the lungs (83). This increase in T cell survival and IFNγ makes infected mice susceptible to infection by decreasing tolerance. Therefore, during *Mtb* infection pushing the expression of IFNγ beyond a protective threshold leads to failed tolerance.

*T cell Metabolism*. While it is possible that results with PD1 are an outlier additional evidence suggests that other alterations to T cell activation may have deleterious effects on tolerating *Mtb*. One recent study found an important role for Cyclophilin D in modulating tolerance to *Mtb* in a T cell dependent manner (84). CyclophilinD (CypD) is a mitochondrial protein that modulates cell death mechanisms such as necrosis (85). Inhibition of CypD in macrophages prevents necrosis and limits *Mtb* replication (86, 87). On this basis, Divangahi and colleagues infected CypD mice, and found that they were highly susceptible to infection (84). However, these mice succumbed to disease with identical burdens of bacteria compared to wild type animals suggesting loss of CypD decreases tolerance to persistent *Mtb* infections. Importantly, the defect in tolerance was not related to differences in cell death and control of *Mtb* replication. Instead CypD was found to regulate a metabolic switch between oxidative phosphorylation and glycolysis in T cells. In the absence of CypD, T cells produced more ROS that drove glycolytic flux, leading to enhanced activation and cytokine production. This critical change in the central metabolism of T cells dramatically reduced the tolerance CypD animals to persistent *Mtb* infection.

Taken together the findings that increasing T cell numbers and enhancing their function in the lungs of *Mtb* infected animals reduces tolerance is compelling. We can no longer pursue the development of therapeutics or vaccines that simply drive more activated T cells and more IFNγ production without considering the very real possibility of deleterious effects. Mammalian hosts have clearly evolved an important balance between antimicrobial resistance strategies and tolerance mechanisms to survive persistent infections that must be more adequately evaluated in our research as we pursue more effective *Mtb* treatment strategies.

### Outlook

The studies discussed above suggest a critical role for the regulation of inflammatory cascades in tolerance to persistent Mtb infection, and highlights a number of well-studied pathways in this process. It seems clear that macrophages integrate metabolic and innate immune signals with those derived from T cells to control the extent of inflammatory tissue damage. While these pathways are important determinants of disease progression, they likely represent a small fraction of the mechanisms that contribute to tolerance. Our current understanding of TB tolerance is focused largely on immunological factors with an already appreciated protective role in the mouse model of TB. However, in simpler model systems, it is clear that a wide variety of functions involved in tissue repair, systemic metabolism, and energy utilization also play an important role. Furthermore, it is clear that bacterial factors interact with the immune system to regulate tolerance, and a number of Mtb genes have been found to alter immunopathology without affecting bacterial fitness (88, 89). Developing models for TB where these diverse tolerance pathways can be observed and dissected represents a major challenge for the future.

While our understanding of tolerance generally lags far behind our knowledge of resistance mechanisms, the examples described above highlight the importance of continued research. While antibiotics are generally effective for uncomplicated Mtb infections, several particularly serious and/or long-term sequelae of Mtb infection can be attributed to defects in tolerance. These complications include acute failures of tolerance, such as meningitis and TB-IRIS, as well as the long-term tissue damage and decreased lung function that generally follows infection. Understanding the processes involved in damage and repair will likely produce more effective therapies.

## Author contributions

AO and CS conceived of and wrote the article together.

### Conflict of interest statement

The authors declare that the research was conducted in the absence of any commercial or financial relationships that could be construed as a potential conflict of interest. The reviewer, MB, and handling editor declared their shared affiliation at the time of the review.
